# Frequency and Characteristics of Infections Caused by Extended-Spectrum Beta-Lactamase-Producing Organisms in Neonates: A Prospective Cohort Study

**DOI:** 10.1155/2013/756209

**Published:** 2013-09-24

**Authors:** Nandini Vijayakanthi, Dheeraj Bahl, Nirmaljit Kaur, Arti Maria, Nand Kishore Dubey

**Affiliations:** ^1^Department of Paediatrics & Neonatology, PGIMER & Associated Dr. Ram Manohar Lohia Hospital, Baba Kharag Singh Marg, New Delhi 110001, India; ^2^Department of Microbilogy, PGIMER & Associated Dr. Ram Manohar Lohia Hospital, Baba Kharag Singh Marg, New Delhi 110001, India

## Abstract

This prospective cohort study was conducted to determine the frequency of infections caused by extended-spectrum beta-lactamase- (ESBL-) producing organisms, various bacteria producing ESBL, antibiotic susceptibility of these organisms, and the risk factors associated with these infections in a neonatal intensive care unit in a tertiary care hospital in North India. Of the 150 neonates enrolled in the study, 47 culture-positive neonates were included in the study cohort and were divided into two groups: ESBL-positive (8 neonates) and ESBL-negative (39 neonates) cohorts. Various organisms were isolated from 72 culture samples in these 47 neonates. Of these, 10 culture samples grew ESBL-positive organisms and 62 samples grew ESBL-negative organisms. The frequency of ESBL-producing organisms was found to be 5.3%. ESBL infection incidence densities were found to be 3.4 per 1000 patient-days. *Klebsiella* (60%) was the most common organism producing ESBL followed by *Escherichia coli* (30%) and *Pseudomonas* (10%). Eighty percent of the ESBL-producing organisms were sensitive to piperacillin-tazobactam. Risk factors found significant by univariate analysis (*P* < 0.05) were preterm, low birthweight, perinatal asphyxia, respiratory distress syndrome, anaemia, metabolic acidosis, prolonged mechanical ventilation (>7 days), length of hospitalization, length of level 3 stay, prior antibiotic use, central venous catheter duration, peripherally inserted central venous catheter duration, and total parenteral nutrition duration. Factors that retained significance in the logistic regression model were duration of hospital stay (adjusted OR: 0.958, CI: 0.920–0.997, and *P* value = 0.037) and gestational age (adjusted OR: 1.39, CI: 1.037–1.865, and *P* value = 0.028). There was no significant difference in the mortality between the two groups.

## 1. Introduction

Broad spectrum penicillins and first-generation cephalosporins remained the first line of defense for nearly 20 years, before resistance to them by beta-lactamases produced by gram-negative bacilli was found to be a serious threat to the common infections prevalent in community and hospital settings [1]. In a few years, cephalosporin-resistant *Klebsiella* species were found among the clinical isolates and the mechanism of this resistance was the production of extended-spectrum beta-lactamase (ESBL) [2].

The first ESBL isolates were discovered in Western Europe in the mid-1980s [3]. ESBLs are plasmid mediated beta-lactamases capable of hydrolyzing and inactivating extended spectrum beta-lactams with an oxyimino side chain like cephalosporins (cefotaxime, ceftriaxone, and ceftazidime) and oxyimino-monobactam (aztreonam). They have no detectable activity against cephamycins and carbapenems [4]. ESBLs are most commonly found in *Klebsiella* species and *Escherichia coli,* but they have also been detected in *Enterobacter* species, *Salmonella* species, *Morganella morganii*, *Proteus mirabilis*, *Serratia marcescens,* and *Pseudomonas aeruginosa* [5]. 

Major risk factors for infection with ESBL-producing organisms are widespread use of third-generation cephalosporins, prolonged intensive care unit (ICU) or hospital stay, instrumentation and catheterization [2]. Patients with septicemia due to ESBL-producing organisms had a significantly higher fatality rate than those with non-ESBL-producing isolates [6]. A recent report from the Infectious Diseases Society of America listed ESBL-producing Klebsiella species and *Escherichia coli* as two of the six drug-resistant microbes to which new therapies are urgently needed [7]. Due to the increasing importance of multiresistant ESBL-producing *Escherichia coli* in the community, clinicians should be aware of the potential of treatment failures associated with serious infections caused by these bacteria [8]. The emergence of ESBL-producing *E. coli* infections in nonhospitalized patients has been recently described in several countries [9–11].

In neonates (0–28 days) ESBL-producing Klebsiella *pneumoniae* is an important cause of nosocomial infections [4]. However, limited information is available on these infections in children especially neonates. The present study is directed to determine the frequency of infections caused by ESBL-producing organisms, the various bacteria producing ESBL, the antibiotic susceptibility of these organisms, and the risk factors associated with these infections in a neonatal intensive care unit in a tertiary care hospital in North India.

## 2. Materials and Methods

### 2.1. Setting

This prospective cohort study was conducted in the tertiary-level referral and inborn neonatal units of the Department of Pediatrics and Neonatology and the Department of Microbiology at Postgraduate Institute of Medical Education and Research and associated Dr. Ram Manohar Lohia Hospital, New Delhi, India, during December 2009–November 2010. Written informed consent was obtained for each subject from the parents. The study was approved by the institute's Ethics Committee.

### 2.2. Study Population

All patients with suspected neonatal sepsis admitted to the referral and inborn neonatal units of Dr. Ram Manohar Lohia Hospital, New Delhi, were included in the study. Suspected neonatal sepsis was defined as the presence of two or more of the following:

- neonates with two or more risk factors [12, 13], see also Table 1 in Supplementary Material available online at (http://dx.doi.org/10.1155/2013/756209),
- neonates with clinical features suggestive of sepsis [14], see also Table 2.1 and 2.2 in Supplementary Material,
- neonates with positive sepsis screen [15, 16], see also Table 3 in Supplementary Material.

Neonates in whom consent was denied and neonates in whom bacterial culture grew a mixture of organisms were excluded from the study.

### 2.3. Data Collection

The following clinical samples were obtained from the suspected cases of neonatal sepsis during the study period as directed by their clinical condition as and where relevant: blood/urine/cerebrospinal fluid/stool/pus/peripheral long line catheter tip/central venous catheter tip/endotracheal tube tip/tracheal aspirate/bronchoalveolar lavage/pleural tap fluid/pericardial tap fluid/ascitic tap fluid. The samples were subjected to standard microbiological methods to isolate and identify the organism, to find the antibiotic susceptibility patterns, and to detect the ESBL-producing organisms.

Information regarding the patient, symptoms, signs, diagnosis, antibiotic usage, interventions, ICU admissions, and outcome was obtained in a semistructured pro forma. Both ESBL-positive and ESBL-negative cohorts were followed up till one of the following end points: discharge, death, or left against medical advice

### 2.4. Methods

Antimicrobial susceptibility testing of all isolates was performed by Kirby Bauer disk diffusion method. In this method, the inoculums were adjusted to the turbidity of a 0.5 McFarland standard and swabbed onto the surface of a Muller-Hinton agar plate. After putting the disks onto the inoculated plates, the plates were incubated at 37°C for 24 hours. Antibiotic potency of the discs was standardized against the reference strain. All susceptibility results were interpreted according to the CLSI (Clinical and Laboratory Standards Institute). The following antimicrobial agents were used for susceptibility testing: ampicillin (A), amikacin (Ak), aztreonam (Ao), ceftazidime (Ca), ceftazidime-clavulanic acid (Cac), cefotaxime (Ce), ceftriaxone (Ci), ciprofloxacin (Cf), cotrimoxazole (Co), cefepime (Cpm), chloramphenicol (C), gentamicin (G), meropenem (Mr), nalidixic acid (Na), nitrofurantoin (Nf), netilmicin (Nt), norfloxacin (Nx), ofloxacin (Of), and piperacillin-tazobactam (Pt). For detection of ESBL production, modified double-disk test was performed as a screening test. ESBL production and susceptibility to antimicrobial agents were detected on the same plate. Susceptibility testing was performed as previously described. Disks containing ceftazidime alone and a combination of clavulanic acid and ceftazidime were placed in a distance of 25 mm (centre to centre). The zones of inhibition for ceftazidime alone and ceftazidime plus clavulanic acid were compared. An increase in zone diameter of 5 mm in the presence of clavulanic acid indicated the presence of ESBL in the test organisms. Automated identification system (Microscan Walkaway, from Siemens) was used for reconfirmation of ESBL production.

### 2.5. Statistical Analysis

Assuming the percentage of neonates with ESBL infections to be 15% in suspected cases of neonatal sepsis admitted to this hospital, with power of 80% with a confidence level of 90%, the minimum sample size needed was 118 cases and with a confidence level of 95%, the minimum sample size needed was 142. A total of 150 cases were enrolled in the study during the study period. The frequency of infections caused by ESBL-producing organisms was reported as the number of infections per 100 neonates with suspect sepsis. ESBL infection *incidence densities* were reported as *the number of infections per 1000* patient days. Categorical variables were reported as numbers with proportion. Continuous variables were reported as mean with standard deviation or median with interquartile range. The association of the study variables with infections caused by ESBL-producing and non-ESBL-producing organisms was tested by univariate analysis. Continuous variables that were normally distributed were analyzed using Student's *t*-test, and the continuous variables that were not normally distributed were analyzed using Wilcoxon rank sum test. The Chi-square or Fisher exact test was used to compare categorical variables. All variables that were found to be statistically significant (*P* value less than 0.05), as a risk factor for infections produced by ESBL-producing organisms, by univariate analysis, were further analyzed by using a multivariate logistic regression model. Statistical software package STATA 11 (College Station, TX, USA) was used for the data analysis. 

## 3. Results

**Figure 1:**
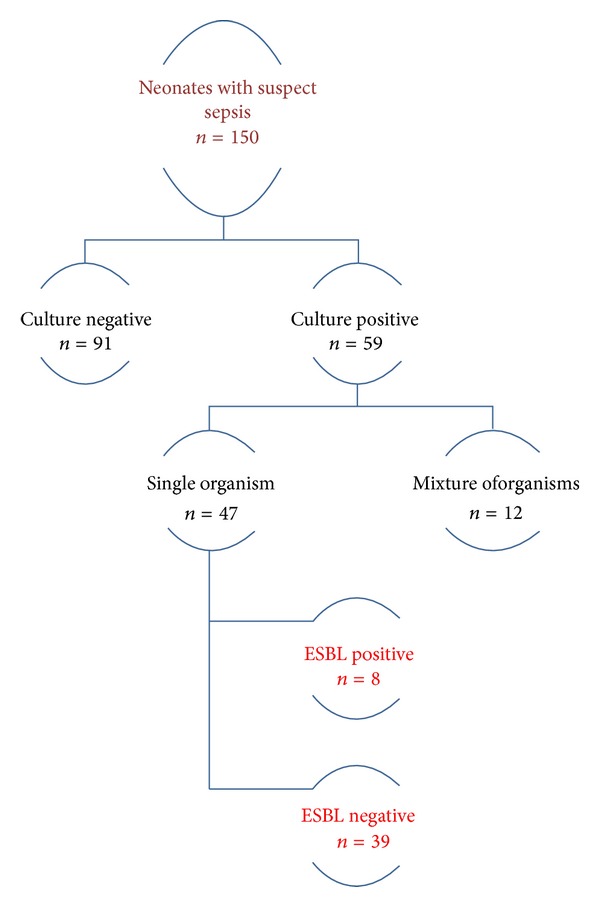
Profile of the study cohort.

A total of 150 neonates with suspect sepsis were enrolled in the study during the period of December 2009–November 2010. Culture-positive gram-negative organisms were found in 59 neonates. Of these mixture of organisms was found in 12 neonates and was excluded from the study. Hence, a total of 47 culture-positive neonates were included in the study cohort. The study cohort was further divided into two cohorts—8 neonates with ESBL-positive cultures and 39 neonates with ESBL-negative cultures (Figure 1). The frequency of ESBL-producing organisms was found to be 5.3%. ESBL infection incidence densities were found to be 3.4 per 1000 patient-days. The baseline characteristics of both ESBL-positive and -negative cohorts are illustrated in Table 1. 

Various organisms were isolated from 72 culture samples in 47 neonates. Of these, 10 culture samples grew ESBL-positive organisms in 8 neonates and 62 samples grew ESBL-negative organisms in 39 neonates. *Klebsiella* (60%) was the most common organism producing ESBL followed by *Escherichia coli* (30%) and *Pseudomonas* (10%). The sources of various organisms producing ESBL and the various organisms producing ESBL are listed in Tables 2 and 3, respectively.

**Figure 2:**
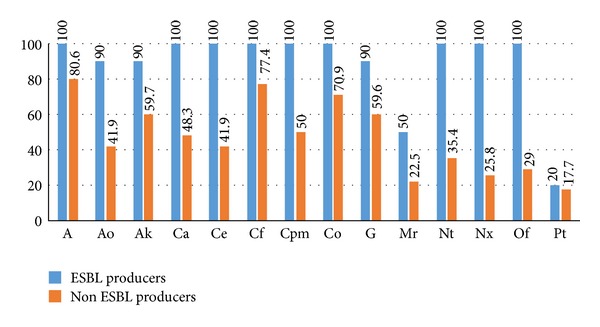
Antimicrobial resistance patterns of the ESBL-producing and non-ESBL-producing organisms. Data are expressed in percentage. ESBL: extended-spectrum beta-lactamase; A: ampicillin; Ao: aztreonam; Ak: amikacin; Ca: ceftazidime; Ce: cefotaxime; Cf: ciprofloxacin; Cpm: cefepime; Co: cotrimoxazole; G: gentamicin; Mr: meropenem; Nt: netilmicin; Of: ofloxacin; Pt: piperacillin-tazobactam.

Prior antibiotic use (ampicillin and cephalosporins) was found to be a significant risk factor associated with ESBL-positive infections (RR: 5.29, CI: 1.21–23.19, and *P* value = 0.011). Eighty percent of ESBL-producing organisms were sensitive to piperacillin-tazobactam, fifty percent to meropenem, and ten percent to aztreonam, amikacin, and gentamicin. The antimicrobial resistance patterns of the ESBL-producing and non-ESBL-producing organisms are given in Figure 2. Analysis of the antimicrobial resistance patterns reveals that ESBL-producing organisms were more resistant to beta-lactam antibiotics compared to non-ESBL-producing organisms. 

Possible risk factors for infection with ESBL-producing organisms are listed in Table 4. Factors that retained significance in the logistic regression model are duration of hospital stay (adjusted OR: 0.958, CI: 0.920–0.997, and *P* value = 0.037) and gestational age (adjusted OR: 1.39, CI: 1.037–1.865, and *P* value = 0.028). 

Among the ESBL-positive cohort, five neonates (62.5 percent) improved, one neonate (12.5 percent) was taken against medical advice, and two neonates (25 percent) expired. Among the ESBL-negative neonates, 32 neonates (82 percent) improved, 1 neonate was taken against medical advice, and 6 neonates (15.4 percent) expired.

## 4. Discussion

Infections caused by ESBL-producing organisms are a significant cause of neonatal morbidity and mortality all over the world mainly attributed to the widespread use of broad-spectrum antibiotics. The incidence of the infections caused by ESBL-producing organisms varies considerably in different geographical situations, from 37% in Latin America, 7% in the United States [17], to 5–56% in various Asian countries [5, 18–21]. In India, a recent study reported a 36.5% and 28.6% prevalence of ESBL-producing *E. coli* and *Klebsiella*, respectively, in neonatal infections [22]. Another similar study from India found the prevalence of ESBL-producing isolates of *E. coli* and *K. pneumonia* to be 22% [23]. The incidence of these infections in the present study (5.3%) is low as compared to the other studies in India

*Klebsiella* (60%) was the most common organism producing ESBL followed by *E. coli* (30%) and *Pseudomonas* (10%). The bacterial spectrum in the present study is comparable to that in other studies [24–26]. Though a few studies have noted the ESBL-producing *Enterobacter* and *Acinetobacter* species, we did not find ESBL production in these organisms [26, 27]. These infections were mostly acquired in the perinatal or neonatal period from the hospital and therefore are multiresistant. Prior antibiotic use (ampicillins and cephalosporins) was found to be a significant risk factor for ESBL production which was specified as one of the major risk factors in other studies [28, 29]. 

The antimicrobial resistance patterns of both ESBL-producing and non-ESBL-producing organisms were comparable with those of other studies [26, 30]. Ampicillin, ciprofloxacin, and cotrimoxazole in general had higher resistance rates among both ESBL-producing and non-ESBL-producing organisms, with the reason being previous widespread use of these antibiotics. In a study, it was described that ESBL-producing *Klebsiella spp*. and ciprofloxacin resistance are closely associated [31]. Recently, the 2008 SMART (Study for Monitoring Antimicrobial Trends) results have emphasized on the alarmingly high (80%) rates of *E. coli* isolates resistant to fluoroquinolones in India [32]. The only hope for treating these infections lies in the carbapenems, piperacillin-tazobactam, and cefoperazone-sulbactam. Piperacillin-tazobactam was effective in 80% of the ESBL producers in the present study which was in unison with a recent study from India where 90% of the ESBL producers were sensitive to piperacillin-tazobactam [33]. Though many other resistance mechanisms for beta-lactam antibiotics like alteration of the penicillin binding proteins, low-affinity penicillin binding proteins, and alteration in the outer membrane permeability have been described in various gram-positive and gram negative cocci, ESBL production remains the main mechanism of resistance in gram-negative bacilli [34].

Preterm and low-birthweight neonates were more prone to infections with ESBL-producing organisms which was consistent with other studies [22, 28, 35], and this is mainly attributed to the immaturity of their immune system; they are also more likely to undergo many interventional procedures [28]. Additionally, we found that, particularly, extremely low-birthweight neonates were more prone to these infections. None of the risk factors for sepsis in mother were found to be associated with ESBL positivity in their neonates. This was consistent with the previous studies [22].

In the present study, respiratory distress syndrome was found to be the major diagnosis in the ESBL-positive neonates. It was the major diagnosis in Huang et al. study, but association was not studied. This association may be attributed to the fact that these neonates are mostly preterm low-birthweight neonates and require interventional procedures and mechanical ventilation [35].

In the present study, the presence of central venous catheters and peripherally inserted central venous catheters (PICC) was significantly associated with ESBL-positive infections which was not a consistent finding and was different in various settings [28, 35–37]. Total parenteral nutrition was reported to be a risk factor for the ESBL-producing organisms in neonates [28, 38]. Our findings confirmed the same.

Endotracheal intubation [22] and prolonged mechanical ventilation [22, 28, 35] were found to be major risk factors for these infections. However, in the present study, though prolonged mechanical ventilation was a significant risk factor, endotracheal intubation was not a risk factor. The presence of the indwelling devices or total parenteral nutrition or endotracheal intubation or mechanical ventilation themselves are not risk factors for sepsis, proper care in the handling of the devices can prevent the infections in these situations. As far as possible the duration of such invasive devices should be minimized and the need for hand washing in the care of such neonates should be emphasized.

Not only the length of hospitalization but also the length of level 3 stay was found to be a risk factor for ESBL infections in neonates which was an additional finding in this study compared to other studies where only the length of hospitalization was found to be a risk factor [22, 28]. This substantiates the fact that level 3 environment plays a key role in the transmission of these organisms as neonates in level 3 are subjected to more invasive procedures and are almost always given higher antibiotics for a prolonged period compared to neonates in level 2 and hence more prone for these infections.

Mortality rates in ESBL-positive infections were higher as compared to the non-ESBL infections [22, 28]. In contrast, it was observed that though the mortality rates were higher for the neonates who had infections by ESBL-producing organisms, statistical significance was not obtained. This may be attributed to the relatively “small sample size” in this study compared to that in the other studies.

During the multivariate logistic regression analysis, the length of hospitalization and gestational age were found to be independent risk factors associated with ESBL-positive infections, which is in harmony with other studies [22, 28]. 

## 5. Conclusion

ESBL test should be routinely done in all culture-positive samples growing gram-negative organisms as the infections by ESBL-producing organisms are a significant problem in neonates. Judicious prescription of antibiotics is recommended as prior use of antibiotics is a significant risk factor for ESBL production. Strict aseptic precautions should be maintained in handling the neonates especially the preterm and low-birthweight neonates. Interventions and duration of hospital stay should be minimized as far as possible. Longitudinal surveillance of the microbial flora and their antibiotic sensitivity pattern should be done in every hospital periodically to know the existing flora and for appropriate management of the infection by these organisms.

## Supplementary Material

Table-1: Risk factors for early onset sepsis
Table-2.1: Clinical features suggestive of sepsis
Table-2.2 :Specific features
Table-3: Sepsis Screen

## Figures and Tables

**Table 1:** Baseline characteristics of the study cohorts.

Characteristics	Neonates with acquisition of infections with ESBL-producing organisms (*n* = 8)	Neonates with acquisition of infections with non-ESBL-producing organisms (*n* = 39)
Median age in days at onset of sepsis (IQR)	1 (1–7)	3 (3–9)
Mean gestational age in weeks (SD)	29 (3.2)	37 (4.2)
Gender		
Male (%)	7 (88)	29 (75)
Female (%)	1 (12)	10 (25)
Birthweight (%)		
<1000 g	3 (38)	2 (5)
1000–1499 g	3 (38)	5 (13)
1500–2499 g	2 (25)	17 (44)
≥2500 g	0 (0)	15 (39)
Preterm delivery (%)	7 (88)	13 (33)
Place of birth (%)		
Inborn	1 (12)	2 (5)
Outborn	7 (88)	37 (95)
Hospital	8 (100)	30 (77)
Mode of delivery (%)		
Vaginal delivery	4 (50)	26 (67)
Cesarean	3 (38)	12 (30)
Assisted delivery	0 (0)	1 (3)
Breech	1 (25)	0 (0)
Meconium stained amniotic fluid (%)	1 (25)	14 (36)
Risk factors for sepsis (%)	3 (38%)	14 (36)
Clinical signs of sepsis (%)	5 (63)	30 (77)
Positive sepsis screen (%)	3 (38)	19 (49)
Early onset sepsis (%)	5 (63)	23 (59)
Late onset sepsis (%)	3 (38)	16 (41)
Median days of hospital stay (IQR)	61 (39–91)	19 (11–21)
Median days of level 3 stay (IQR)	54 (35–77)	11 (7–22)
Median days of level 2 stay (IQR)	8 (4–11)	6 (2–8)
Outcome (%)		
Expired	2 (25)	6 (15)

ESBL: extended-spectrum beta-lactamase; IQR: interquartile range; SD: standard deviation.

**Table 2:** Sources of various isolates in infected neonates.

	ESBL-positive isolates (*n* = 10)	ESBL-negative isolates (*n* = 62)
Blood specimens	2 (20)	13 (21)
Endotracheal tube tip/aspirate specimens	4 (40)	24 (39)
Cerebrospinal fluid specimens	0 (0)	1 (2)
Urine	0 (0)	3 (5)
PICC tip/UVC tip	2 (20)	10 (16)
Surgical wound swabs	2 (20)	11 (18)

ESBL: extended-spectrum Beta-lactamase; PICC: peripherally inserted central venous catheter; UVC: umbilical venous catheter.

Data are expressed as numbers (%).

**Table 3:** Organisms isolated from various culture specimens.

Organisms	ESBL-positive cultures (*n* = 10)	ESBL-negative cultures (*n* = 62)
*Klebsiella pneumoniae *	6 (60)	17 (27)
*Escherichia coli *	3 (30)	4 (6)
*Pseudomonas *	1 (10)	3 (5)
*Acinetobacter *	0 (0)	28 (45)
*Enterobacter *	0 (0)	8 (13)
*Citrobacter *	0 (0)	2 (3)

ESBL: extended-spectrum beta-lactamase.

Data are expressed as numbers (%).

**Table 4:** Univariate analysis of various risk factors for infections with ESBL-producing organisms.

Variable	Neonates with acquisition of infections with ESBL-producing organisms (*n* = 8)	Neonates with acquisition of infections with non-ESBL-producing organisms (*n* = 39)	*P* value	Relative risk (95% CI)
Birthweight (g)	1088 (825)	2253 (745)	0.001	—
Gestational age (weeks)	29 (3.24)	37 (4.15)	0.001	—
Birthweight < 1000 g (ELBW)	3 (38)	2 (11)	0.028	5.04 (0.94–150.82)
Preterm	7 (88)	13 (33)	0.005	0.11 (0.01–0.79)
Perinatal asphyxia	7 (88)	16 (41)	0.017	7.30 (0.97–54.83)
Mechanical ventilation > 7 days	5 (63)	10 (26)	0.041	0.28 (0.08–1.03)
Anemia	3 (38)	4 (10)	0.049	5.25 (0.67–43.39)
Metabolic acidosis	3 (38)	3 (8)	0.021	4.10 (1.30–12.91)
Respiratory distress syndrome	5 (63)	3 (8)	0.001	8.13 (2.42–27.32)
Central venous catheter duration (days)	12 (5.4)	2.4 (4.3)	0.001	—
PICC duration (days)	29 (13–35)	7 (0–12)	0.006	—
TPN duration (days)	26 (16–31)	7 (0–12)	0.001	—
Duration of hospital stay (days)	61 (39–91)	19 (11–21)	0.002	—
Duration of level 3 stay (days)	54 (35–77)	11 (7–22)	0.001	—

ESBL: extended-spectrum beta-lactamase; CI: Confidence interval; PICC: peripherally inserted central venous catheter; TPN: total parenteral nutrition.

Univariate analysis of risk factors: only those with *P* value < 0.05 are shown.

Data are expressed as numbers (%), mean (standard deviation), and median (interquartile range).
