# Supplementary material for: Frequency and Characteristics of Infections Caused by Extended-Spectrum Beta-Lactamase-Producing Organisms in Neonates: A Prospective Cohort Study
Source: Biomed Res Int. 2013 Sep 24;2013:756209. doi: 10.1155/2013/756209 (PMC3794505; doi:10.1155/2013/756209)
Supplement: Supplementary file 1 — Table-1: Risk factors for early onset sepsis Table-2.1: Clinical features suggestive of sepsis Table-2.2 :Specific features Table-3: Sepsis Screen [file 756209.f1.pdf]

Supplementary material

Table-1 : Risk factors for early onset sepsis

- Rupture of membranes (>24 hours)
- Foul smelling and / or meconuim stained liquor aminii
- Febrile illness in the mother with the evidence of bacterial infection ,within two weeks prior to delivery
- Single unclean or >3 sterile vaginal examinations during labor
- Clinical features of chorioamnionitis such as fever, abdominal pain, tachycardia, leucocytosis, uterine tenderness, foul smelling liquor.
- Low birth weight (<2500 grams) or prematurity
- Perinatal asphyxia (Apgar score <4 at 1 minute of age)

Table-2.1: Clinical features suggestive of sepsis

Non-specific features

- Hypothermia or fever;
- Lethargy, poor cry, refusal to feed;
- Poor perfusion, prolonged capillary refill time;
- Hypotonia, absent neonatal reflexes;
- Bradycardia/Tachycardia;
- Respiratroy distress, apnea, gasping respiration;
- Hypoglycemia/hyperglycemia
- Metabollic acidosis

Table-2.2 :Specific features

• Bulging anterior fontanelle	• Abdominal distension'
• Vacant stare	• Vomiting
• High pitched cry	• Diarrhea
• Excessive irritability	• Bleeding
• Altered sensorium	• Petechiae

• Seizures<br>• Neck retraction<br>• Hypotension<br>• Shock<br>• Jaundice with high colored urine	• Sclerema<br>• Multiple pustules /abscess/mottling<br>• Non passage of urine >12 hours<br>• Umbilical sepsis in the form of redness/discharge

Presence of any one of the above features was considered suggestive of sepsis

Table-3: Sepsis Screen

**Parameters**	**Abnormal value**
Total Leucocyte count	5000/cu.mm
Absolute neutrophil count	Low counts as per Monroe chart
Immature/Total neutrophil ratio	>0.2
Micro ESR	Age in days + 3
C-Reactive Protein	Positive (>1mg/dl)

Positive sepsis screen included at least two abnormal parameters.
